# Trust, Identity, and Public-Sphere Pro-environmental Behavior in China: An Extended Attitude-Behavior-Context Theory

**DOI:** 10.3389/fpsyg.2022.919578

**Published:** 2022-06-24

**Authors:** Yunfeng Xing, Mengqi Li, Yuanhong Liao

**Affiliations:** College of Economics and Management, China Agricultural University, Beijing, China

**Keywords:** trust, identity, public-sphere pro-environmental behavior, attitude-behavior-context theory, mechanism

## Abstract

Changing human behavior is critical to mitigating the increasingly severe environmental harm. Although numerous studies focus on private-sphere or generalized pro-environmental behavior (PEB), relatively little research examines explicitly public-sphere PEB from a collective action perspective. This study incorporates trust and identity into the Attitude-Behavior-Context (ABC) theory to investigate Chinese residents’ participation in public-sphere PEB. Primary data collected from 648 residents in China tested the model empirically. The results indicate that social trust, environmentalist self-identity, and politicized identity positively predict public-sphere PEB and that institutional trust positively impacts non-activist behaviors but negatively relates to environmental activism. There is also evidence that trust and identity are moderators of attitude and public-sphere PEB. Specifically, social trust and environmentalist self-identity strengthen the effect of attitude on public-sphere PEB. Politicized identity increases the impact of attitude on environmental activism but not on non-activist behaviors, and there is no significant moderating effect of institutional trust. The findings deepen the understanding of public-sphere PEB and make more targeted policies accordingly.

## Introduction

With the development of industrial civilization, many environmental problems, such as climate change, air pollution, and resource depletion, are increasingly severe (Carfora et al., 2017). Changing human behavior can manage many of these problems. According to Steg and Vlek (2009), pro-environmental behavior (PEB) are activities that cause minimal damage to the environment or benefit the natural environment. Stern (2000) identified four distinct types of PEBs, specifically, environmental activism, non-activist behaviors in the public-sphere, private-sphere environmentalism, and other environmentally significant behaviors. Among them, environmental activism and non-activist behaviors are two typical public-sphere PEBs. Public-sphere PEB is considered to be the environmental behavior with characteristics of “collective action,” such as making environmental complaints, participating in environmental protection activities and organizations, and accepting environmental protection policies (Wan and Du, 2022). In addition, participating in environmental social movements, donating money, conducting demonstrations, and signing petitions are also significant forms of public-sphere PEB (Stern, 2000; Hadler and Haller, 2011). In contrast, private-sphere PEBs focus on ecological protection behaviors in citizens’ personal lives. Stern (2000) proposed that private-sphere PEB includes the purchase, use, and disposal of household or personal products that have an impact on the environment. For instance, the use of new energy vehicles, public transportation, recycling, green consumption, and energy-saving behaviors are common private-sphere PEBs (Hunter et al., 2004; Ertz et al., 2016).

Numerous studies focused on private-sphere PEBs and found that intrapersonal factors, such as personal beliefs, attitudes, or identity, are critical factors for private-sphere PEBs (van der Werff and Steg, 2016; Gkargkavouzi et al., 2019). However, only a few studies have explored the determinants of a specific type of public-sphere PEB. For instance, Song et al. (2019) verified that trust is a significant influencing factor of environmental citizenship behavior. Moreover, Dono et al. (2010) proposed that social identity has an indirect effect on environmental activism. Besides, other determinants of environmental activism were also examined, such as environmental attitude, perceived ecological risks, government responsiveness and transparency, individual resources, and willingness to contribute (Sguin et al., 1998; Marquart-Pyatt, 2012; Peng and Zhong, 2020). In addition, some other research explore a range of behaviors under the conceptual umbrella of PEB. For example, Ertz et al. (2016) examined the role of contextual factors on public- and private-sphere PEBs based on the same framework. Similarly, Mi et al. (2021) investigated how COVID-19 emergency cognition influences PEB intentions in the household, workplace, and public sphere.

However, the development of PEB in the private and public sphere is quite different. Dalton (2015) investigated PEB in eight countries from 1993 to 2010 and claims that the participation rate of sorting recyclables increased from 62 to 83% while that of public-sphere PEB dropped markedly. Moreover, the variables explaining private-sphere PEB are relatively poor predictors of public-sphere PEB (Dono et al., 2010; Alisat and Riemer, 2015), although some research highlighted that contextual factors (Fritsche et al., 2018), group-related variables (Schulte et al., 2020), and social identity (van Zomeren et al., 2011) are critical determinants of public-sphere PEB. Public-sphere PEB has not been well understood (Dono et al., 2010), and less work focuses on its collective action nature of it. Thus, developing a more precise, specific, and group-process orientation framework to explore the determinants of public-sphere PEB is a vital research requirement.

Therefore, the purposes of this research are as follows: (1) to explore the impact of trust (social trust and institutional trust) and identity (environmentalist self-identity and politicized identity) on public-sphere PEB (environmental activism and non-activist behaviors) and (2) to examine the moderating role of trust and identity on the relationship between attitude and two types of public-sphere PEB.

## Literature Review and Hypothesis Development

### Public-Sphere Pro-environmental Behavior

People can engage in both the private and public-sphere PEB to mitigate the negative impact on the environment (Tam, 2020; Cao and Chen, 2021). To date, however, far too little attention has been paid to public-sphere PEB. Most prior research focused on private-sphere PEB (Wynes and Nicholas, 2017). The others analyzed private-sphere and public-sphere PEB in the same framework (Ertz et al., 2016; Mi et al., 2021), which merges the differences between the two types of PEB. These models relatively poorly explain the public-sphere PEB compared to private-sphere PEB (Dono et al., 2010). Only a few studies examined specific public-sphere PEBs, such as environmental activism (Schmitt et al., 2019) or environmental citizenship behavior (Song et al., 2019). Therefore, the existing research has not captured the whole picture of public-sphere PEB, and there has been no detailed investigation of specific determinants of public-sphere PEBs.

This study focuses on the role of trust and identity in public-sphere PEBs, trying to reveal the logic of public-sphere PEB from a collective action perspective. We divided public-sphere PEB into environmental activism and non-activist behaviors, following Stern (2000). Environmental activism refers to the engagement in or support of environmental movements aiming to fight environmental injustices (Sguin et al., 1998), especially in the political realm (Dalton, 2015), for instance, participation in political actions (Stern et al., 1999), petitions, and demonstrations on environmental issues (Stern, 2000). Non-activist behaviors can be understood as people expressing their environmental needs, concerns, and interests through institutional channels and supporting or accepting public policies (Song et al., 2019).

### Attitude-Behavior-Context Theory

The Attitude-Behavior-Context (ABC) theory is original from the research of Stern and Oskamp (1987). They proposed that PEB results from a series of causal relationships between external and internal factors (Stern and Oskamp, 1987). Guagnano et al. (1995) further pointed out that the inner environmental attitude (A) and external contextual factors (C) and their interactions determine PEB. The ABC theory widely applies to the study of PEB, such as climate warming, green consumption, and waste recycling (Ertz et al., 2016; Huang, 2016).

Attitude refers to a degree of preference or disfavor for a specific entity (Eagly and Chaiken, 1993). Pro-environmental attitude has been verified as one of the essential predictors of PEB (Duarte et al., 2017). For example, Verplanken and Holland (2002) believed that individuals need to change their intrinsic motivation, ecological values, and related attitudes for engaging in PEB. In addition, people with positive attitudes are more likely to participate in environmental organizations (Malik and Singhal, 2017). Based on the analysis above, we propose the following hypotheses:

H1a: Attitude has a positive impact on environmental activism.

H1b: Attitude has a positive impact on non-activist behaviors.

Some scholars argued that the existing models of environmental activism focus exclusively on individuals’ characteristics but neglect contextual and interpersonal factors (Wakefield et al., 2006), while the contextual factors were more important than individual characteristics for public-sphere PEB (Dono et al., 2010). In addition to attitudinal variables, the ABC theory complements contextual factors and their interaction to explain PEB (Guagnano et al., 1995). Both objective and subjective factors can be regarded as contextual factors. The former includes monetary incentives, costs, regulations, or public policy; the latter includes some subjectively perceived factors, such as perceived resources availability (Ollie et al., 2001). Therefore, we considered social norms and contextual constraints as two representatives of contextual factors in this study.

Contextual constraints refer to the objective facility and conditions that impede PEB, such as the extra effort, time, and cost of PEB (Young et al., 2010; Grimmer et al., 2015). When the context of performing a particular behavior is complex, inconvenient, or expensive, the behavior does not necessarily occur even under the influence of personal attitude (Stern, 2000). When people perceive that public-sphere PEB may require more time (Dubuisson-Quellier and Lamine, 2008), more resources (Clark et al., 2003), or higher power, their willingness to participate reduces (Grimmer et al., 2015). Thus, we propose the following hypotheses:

H2a: Contextual constraints have a negative impact on environmental activism.

H2b: Contextual constraints have a negative impact on non-activist behaviors.

Social norms are the common beliefs held by the general public and behavioral standards that influence their activities (Ostrom, 2000). If individuals conform to environmental social norms, they would be more likely to engage in PEB for various reasons, such as desiring to fit in, gaining social esteem, and avoiding social disapproval (Smith et al., 2012; Farrow and Grolleau, 2017). Many studies showed that social norms positively affect a wide range of PEBs, such as green consumption (Yadav and Pathak, 2016), recycling (Sorkun, 2018), and littering (Shimazu, 2018). Furthermore, public-sphere PEB engagement represents more the nature of collective action than the private-sphere PEB. People face a social dilemma to either participate to maximize the society’s welfare or participate in free ride and benefit from others’ actions. In this context, social norms might align self-interest with collective interests by imposing sanctions on individuals. China is a society with a solid collectivistic culture (Wang M. et al., 2021), where people may attach great importance to social norms (Eom et al., 2016). Therefore, we proposed the following hypotheses:

H3a: Social norms have a positive impact on environmental activism.

H3b: Social norms have a positive impact on non-activist behaviors.

### Trust

Trust is an intention to accept vulnerability due to positive expectations of the preferences or actions of others (Rousseau et al., 1998), which could alleviate social dilemmas and build conditional cooperation in PEB (Tam and Chan, 2018). Trust can be divided into social trust and institutional trust (Jones et al., 2011).

Social trust, also named generalized trust, refers to trust in others within a society, reflecting collective social bonds within a society (Smith and Mayer, 2018). As such, the level of trust in strangers or cross-group members in the same community measures social trust (Carattini et al., 2015). Prior research provides evidence that social trust contributes to public-sphere PEB. For example, Smith and Mayer (2018) found that social trust and reciprocal expectations are essential when solving collective action issues like climate change. Wagner and Fernandez-Gimenez (2008) pointed out that social trust comes from the common perception of similar behaviors taken by groups to protect public goods, which can effectively promote public-sphere PEB. Furthermore, Tam and Chan (2018) proved that generalized trust had a more robust effect on public-sphere PEB than private-sphere PEB.

Institutional trust, or political trust, refers to trust in institutions, such as the government, the legal system, or other management agencies (Wynveen and Sutton, 2015; Smith and Mayer, 2018). An institution’s transparency, competence, objectivity, and fairness are the critical elements of institutional trust (Giddens, 1990). Arbuckle et al. (2013) and Nunkoo et al. (2013) demonstrated that residents’ institutional trust level determines whether they support public environmental policies. However, Mark (2002) argued that neither political trust nor social trust significantly affects environmental activism. Thus, we propose the following hypotheses:

H4a: Social trust has a positive impact on environmental activism.

H4b: Social trust has a positive impact on non-activist behaviors.

H5a: Institutional trust has a positive impact on environmental activism.

H5b: Institutional trust has a positive impact on non-activist behaviors.

### Identity

Individuals can construct various identities based on their demographic, characteristics, social roles, and group affiliations (Ashforth and Mael, 1989). We focused on environmentalist self-identity (EI) and politicized identity in this study based on identity theory (Stryker, 1968) and social identity theory (Tajfel, 1978).

Identity theory clarifies the logical relationship between self-identity and behavior (Stryker, 1968). EI refers to a durable sense of oneself as an environmentally friendly person (Whitmarsh and O’Neill’s, 2010). According to identity theory, an individual would adopt some behavior to validate ones’ self-concept or avoid the conflict with role-inappropriate behavior (Clayton, 2012). The more essential and salient an identity, the greater the probability of a role-consistent action. EI may encourage individuals to engage in PEB (Carfora et al., 2017). Specifically, when people have a stronger EI, they will feel a more outstanding moral obligation (van der Werff et al., 2013a) or sacrifice their interests to a certain extent (van der Werff et al., 2013b) to take pro-environmental actions. Particularly, Fielding et al. (2008) demonstrated that EI was a stronger predictor of environmental activism intention. Thus, we propose as following:

H6a: EI has a positive impact on environmental activism.

H6b: EI has a positive impact on non-activist behaviors.

Social identity theory captures that individuals evaluate themselves as members of specific groups and discriminate against outsiders (Tajfel, 1978). Some studies found that social identity is significantly related to public-sphere PEB (Dono et al., 2010), such as environmental activism (Brunsting and Postmes, 2002), protest participation (Klandermans, 2002), and union participation (Veenstra and Haslam, 2000).

As a specific social identity, politicized identity refers to a durable sense of oneself as an activist or identification with a social movement (van Zomeren et al., 2008). A few studies suggested that politicized identity often affects individuals’ perceptions and decisions (Fisher and Sakaluk, 2019). For example, people might justify engaging in public-sphere PEB based on their group’s perceived attributes (Milner et al., 2019). Moreover, more specific to collective action, politicized identity could inspire a stronger internal obligation to participate in a social movement. For instance, Fielding et al. (2008) verified that “environmental activist” identity can predict environmental activism. Furthermore, politicized identity is more strongly associated with collective action (van Zomeren et al., 2008) and environmental activist behavior, while EI is more strongly associated with private-sphere PEB (Mackay et al., 2021). Thus, we propose the following hypotheses:

H7b: Politicized identity has a positive impact on environmental activism.

H7b: Politicized identity has a positive impact on non-activist behaviors.

### Moderating Effect of Trust

The existence of the attitude-behavior gap (Babutsidze and Chai, 2018) is usually acknowledged in the context of PEB. Worrying about being exploited by free riders is one reason for this gap, especially for those with a high pro-environmental attitude (Carrington et al., 2014). Social trust was proven as an effective way to narrow the attitude–behavior gap for some reasons. First, social trust provides a good atmosphere for collective actions, such as knowledge and practical experience-sharing behavior (López-Mosquera et al., 2015). This information will make PEB feel more accessible and convenient for people. Second, individuals with high social trust would believe others’ commitment to PEB, which can temper their fear of free riders (Tam and Chan, 2018). Therefore, individuals with high social trust are more likely to translate their pro-environment attitude into actual behavior (Farrow and Grolleau, 2017).

Moreover, individuals with a high institutional trust may believe that the government and agencies have the competence to manage environmental issues (Mark, 2002). These beliefs can also contribute to their attitudes turning into behaviors. It means that, even if two individuals have the same level of pro-environment attitude, the one with higher institutional trust will be more likely to express their environmental advocacy to the government, will be more likely to cooperate with public institutions, and would be more willing to support environmental policies (Caferra et al., 2021). We thus hypothesize the following:

H8a: Social trust strengthens the effect of attitude on environmental activism.

H8b: Social trust strengthens the effect of attitude on non-activist behaviors.

H9a: Institution trust strengthens the effect of attitude on environmental activism.

H9b: Institution trust strengthens the effect of attitude on non-activist behaviors.

### Moderating Effect of Identity

A criticism of previous studies of identity is that they neglect the moderation effects (Carfora et al., 2017). We argue that even though environmental attitude leads some residents to engage in PEBs, not everyone gets involved in the public-sphere PEB. To avoid debates or differences with others, some individuals are reluctant to participate in environmental activism or express their environmental needs and concerns, even though they have environmental attitudes (Bhatti et al., 2020). As EI becomes strong, individuals are more likely to transform their attitudes into actual behaviors because they need some symbolic actions to convey their environmentalist identity and maintain consistency between their behavior and identity. At the same time, the environmental attitude more strongly relates to public-sphere PEB when politicized identity was high. Except for consistency, another explanation might be that people with high politicized identities have more initiative, enthusiasm, and experience in public-sphere PEB. Therefore, they are ready for such possible conflicting situations. The following hypotheses were proposed:

H10a: EI strengthens the effect of attitude on environmental activism.

H10b: EI strengthens the effect of attitude on non-activist behaviors.

H11a: Politicized identity strengthens the effect of attitude on environmental activism.

H11b: Politicized identity strengthens the effect of attitude on non-activist behaviors.

### The Research Framework

**FIGURE 1:**
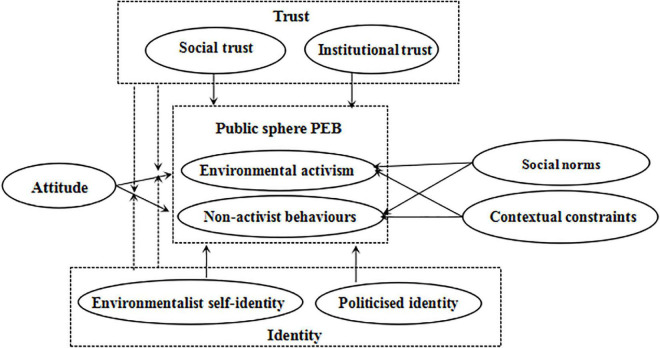
The research framework.

The ABC theory has been specifically developed to predict PEB and proven by numerous studies (Guagnano et al., 1995). For instance, Shi et al. (2019) comprehensively used the ABC theory and the theory of planned behavior (TPB) to explore the influence of psychological factors and policy factors on PM2.5 reduction behavior. Liao and Yang (2022) applied the ABC theory, the TPB theory, and the norm activation model to examine the determinants of PEBs in the private sphere. In addition, some scholars draw on the ABC theory to measure the impact of perceived wealth, perceived power, and perceived busyness as contextual factors on environmental citizenship behavior and private-sphere PEB (Ertz et al., 2016). Therefore, we selected the ABC theory as the basic model. However, compared with private-sphere PEB, public-sphere PEB is more compatible with the collectivist environmental perspective (Clayton and Opotow, 2003). Consequently, other factors were more important than personal variables for public-sphere PEB (Wakefield et al., 2006), such as the perception of trust and identity. As a result, we extended the ABC theory by adding trust and identity factors to explore their roles in public-sphere PEB from a collective action perspective. To better understand the mechanisms, this study also examines the moderating effects of trust and identity. The conceptual framework of this study is shown in Figure 1.

## Data and Methodology

### Sample and Data Collection

The survey was conducted from October to December of 2020 in Beijing, Shanghai, Guangdong, Jiangsu, and Zhejiang, and the target respondents were residents above the age of 18. We believe that the citizens there have an advanced awareness of environmental protection in those most developed regions in China. Furthermore, they are in the north, south, and southeast of China, and the residents there may represent different cultures and different behavioral habits.

A pre-test with a sample of 70 was implemented to test the scales. Then, we entrusted Wenjuanxing to distribute the formal questionnaire. Wenjuanxing^1^ is the most specialized online survey platform in China, with more than 28.7 million registered members (Wang et al., 2019). According to Chinese urban population characteristics, we set gender and age quotas for respondents. The Wenjuanxing platform generated an URL (Uniform Resource Locator) for our questionnaire and distributed it to the eligible members based on our quotas. “Wenjuanxing” has set the screening mechanisms to control the quality of the questionnaires, such as each participant should have a different IP address, and the questionnaire must be completed within 5–15 min. We also excluded questionnaires with too many missing values and with the same answers for five or more consecutive items (Liao, 2021). The respondents who submitted qualified questionnaires were paid about $2.5. As a result, we received 827 finished questionnaires in total, and among them, 648 were valid. Of these 648 respondents, 45.5% were men and 54.5% were women. Most respondents were aged from 31 to 45. Most (69.1%) had a bachelor’s degree. The monthly income was mostly from RMB 5,000 to RMB 10,000 ($705 to $1,410).

### Measures

There were two parts to the questionnaire. The first part was about public-sphere PEB and its potential determinants. The second part examined the demographic characteristics of respondents.

Most items of the constructs were adapted from prior studies. In particular, pro-environmental attitudes were measured using items from the scales of Gao et al. (2017) and Gkargkavouzi et al. (2019). The contextual constraints scale was adapted from Ertz et al. (2016) and Gkargkavouzi et al. (2019). The items for social norms were adapted from the scales of Ling and Xu (2020). Scales for the social trust were revised from the studies of Liu et al. (2014) and Kuo et al. (2021). Items for the political trust were modified from the studies of Caferra et al. (2021) and Kitt et al. (2021). EI (Fielding et al., 2008; Whitmarsh and O’Neill, 2010) and politicized identity (van Zomeren et al., 2008) were developed based on previous research. The constructs above were all measured using five-point Likert scales, where one represented “strongly disagree” and five represented “strongly agree.”

We used eight questions to capture two types of public-sphere PEB. Specifically, four items for environmental activism were modified from the items of Stern (2000), Postmes (2002), and Jiménez-Castillo and Ortega-Egea (2015); four items for non-activist behaviors were adapted from the scales of Dono et al. (2010) and Ertz et al. (2016). We used the following question to measure the frequency of behaviors: What is the percentage of time you did each of the following in the past 5 years? (choose the closest option). The options were as follows: never (1), less than once every 3 years (2), once every 2 or 3 years (3), roughly once a year (4), and more than once a year (5).

### Analytical Techniques

We constructed a structural equation model (SEM) for this study. SmartPLS version 3.0 was used to analyze the data, and the bootstrap resampling method (5,000 resamples) was applied to test the statistical significance of the model. The partial least square (PLS) SEM is an estimation method of component-based and can estimate the simultaneous relationships among multiple latent variables (Jääskeläinen et al., 2020). There are some reasons to use PLS-SEM. First, PLS-SEM does not require a normal distribution of data (Fornell and Bookstein, 1982) and is suitable for the unknown or uncertain distributions data (Hair et al., 2017), such as the data from Likert scales. Second, PLS-SEM is useful for testing the complex investigated model, for example, models with mediation and (or) moderation variables (Hair et al., 2014). Third, new latent variables or new relationships can be introduced flexibly to an established theory in PLS-SEM (Richter et al., 2016), which applies to our study. Consequently, it is a proper choice to test our research model by PLS-SEM.

## Data Analysis and Results

### Common Method Variance

All the items in our investigation were answered by the same interviewee, which may lead to the common method variance (CMV) and endanger the effectiveness of the scale. According to Schwarz et al. (2017), the CMV was evaluated by Harmen’s single factor test. The results informed that four factors appeared in the factor analysis, with the first factor explaining 12.18% of the total variance, far below the 50% threshold value (Harman, 1967). Furthermore, we found no excessive correlations in Table 1, all below the threshold of 0.7 (Tabachnick and Fidell, 2013). The results showed that CMV is not a threat to our data.

**TABLE 1:** Correlations and square roots of AVEs (Fornell–Larcker criterion).

Construct	AT	SN	CC	PI	EI	ST	IT	NA	EA
AT	**0.792**								
SN	0.491	**0.793**							
CC	–0.387	–0.354	**0.862**						
PI	0.58	0.536	–0.408	**0.879**					
EI	0.543	0.596	–0.424	0.703	**0.799**				
ST	0.559	0.608	–0.443	0.602	0.659	**0.832**			
IT	0.458	0.458	–0.335	0.494	0.478	0.507	**0.794**		
NA	0.617	0.574	–0.507	0.627	0.669	0.659	0.538	**0.794**	
EA	0.486	0.527	–0.41	0.557	0.557	0.550	0.299	0.593	**0.771**

*The diagonal (bold) elements are the square roots of AVEs, and the off-diagonal elements are the correlations among constructs.*

*AT, attitude; SN, social norms; CC, contextual constraints; PI, politicized identity; EI, environmentalist self-identity; ST, social trust; IT, institutional trust; NA, non-activist behaviors; EA, environmental activism.*

### The Measurement Model

Composite reliability (CR) and Cronbach’s alpha can measure the reliability (Hair et al., 2014). All of Cronbach’s alpha values and CR values were above the cutoff value of 0.7, indicating the high levels of internal consistency of the scales (Nunnally, 1978).

Convergent validity refers to how the measure of a construct correlates. The convergence validity of the model needs to meet the conditions that the AVE values are above 0.5, and the standardized factor loadings are greater than 0.7 (Hair et al., 2014). As shown in Table 2, all the values are within a reasonable range. Moreover, multicollinearity among the constructs proves not to be a severe concern in this study, as no variance inflation factor exceeded the 2.0 level except CC2.

**TABLE 2:** Reliability and validity tests of the constructs.

Construct	VIF	Items	Standard loadings	Cronbach’sα	CR	AVE
AT	1.375	AT1	0.777	0.703	0.835	0.627
	1.332	AT2	0.785			
	1.417	AT3	0.814			
SN	1.307	SN1	0.759	0.704	0.835	0.628
	1.408	SN2	0.789			
	1.435	SN3	0.828			
CC	1.763	CC2	0.845	0.827	0.896	0.743
	2.025	CC3	0.874			
	1.900	CC4	0.866			
PI	1.422	PI1	0.881	0.705	0.872	0.772
	1.422	PI3	0.877			
EI	1.391	EI2	0.794	0.716	0.841	0.638
	1.359	EI3	0.782			
	1.477	EI4	0.819			
ST	1.579	ST1	0.808	0.779	0.871	0.693
	1.598	ST2	0.838			
	1.657	ST3	0.851			
IT	1.430	PT1	0.813	0.707	0.837	0.631
	1.301	PT2	0.760			
	1.453	PT3	0.808			
NA	1.383	NA1	0.807	0.707	0.836	0.63
	1.354	NA3	0.766			
	1.404	NA4	0.808			
EA	1.729	EA1	0.829	0.775	0.854	0.595
	1.456	EA2	0.781			
	1.575	EA3	0.747			
	1.510	EA4	0.723			

*(1) CR is short for composite reliability; (2) AVE is short for average variance extracted.*

*(3) AT1–EA4 are the items that measured the constructs (see Supplementary Appendix).*

*AT, attitude; SN, social norms; CC, contextual constraints; PI, politicized identity; EI, environmentalist self-identity; ST, social trust; IT, institutional trust; NA, non-activist behaviors; EA, environmental activism.*

The significance of discriminant validity is not only to ensure deterministic results but also to ensure that there is no statistical difference (Henseler et al., 2015). Fornell and Larcker criterion and heterotrait–monotrait (HTMT) ratio are considered to be effective methods for evaluating discriminant validity (Henseler et al., 2015). The Fornell and Larcker standard suggests that a certain variable is supposed to show more variance in comparison with their own items rather than with other variables (Hair et al., 2011). The premise of the discriminant validity is that HTMT values among different constructs are below 0.9 (Henseler et al., 2015). Table 1 provides the results of Fornell and Larcker criterion. Each square root of the AVE value is greater than its highest correlation with other constructs, indicating a good discriminant validity (Degirmenci and Breitner, 2017). Table 3 shows that all the HTMT ratio values except one are lower than the threshold of 0.9 (Ringle et al., 2015). Although the correlation between EI and politicized identity is greater than 0.90, the upper confidence interval is 0.979, which is below 1, indicating no significant concern with discriminant validity (Cao et al., 2021).

**TABLE 3:** Heterotrait–monotrait ratio (HTMT) and confidence interval.

	AT	SN	CC	PS	ES	ST	PT	NA
SN	**0.708** [0.611, 0.797]							
CC	**0.509** [0.416, 0.598]	**0.462** [0.368, 0.553]						
PI	**0.811** [0.717, 0.899]	**0.740** [0.657, 0.822]	**0.527** [0.439, 0.617]					
ES	**0.712** [0.627, 0.793]	**0.804** [0.727, 0.877]	**0.524** [0.432, 0.617]	**0.914** [0.848, 0.979]				
ST	**0.755** [0.678, 0.825]	**0.818** [0.746, 0.884]	**0.554** [0.474, 0.633]	**0.800** [0.729, 0.869]	**0.847** [0.778, 0.912]			
IT	**0.651** [0.551, 0.751]	**0.651** [0.572, 0.729]	**0.439** [0.350, 0.528]	**0.699** [0.613, 0.784]	**0.627** [0.540, 0.718]	**0.683** [0.609, 0.756]		
NA	**0.708** [0.792, 0.946]	**0.811** [0.723, 0.894]	**0.661** [0.585, 0.738]	**0.868** [0.790, 0.941]	**0.893** [0.802, 0.980]	**0.881** [0.807, 0.953]	**0.757** [0.685, 0.830]	
EA	**0.649** [0.574, 0.724]	**0.697** [0.614, 0.774]	**0.488** [0.406, 0.567]	**0.717** [0.636, 0.790]	**0.693** [0.599, 0.778]	**0.690** [0.618, 0.756]	**0.385** [0.298, 0.470]	**0.784** [0.712, 0.851]

*The bold elements are the correlations among constructs and the confidence interval of the value is in parentheses. AT, attitude; SN, social norms; CC, contextual constraints; PI, politicized identity; EI, environmentalist self-identity; ST, social trust; IT, institutional trust; NA, non-activist behaviors; EA, environmental activism.*

### The Structural Model

#### Predictive Relevance of the Model

Cross-validated redundancy and determination coefficient can effectively evaluate the predictive relevance of the model (Hair et al., 2011). *R*^2^ is the primary measure of the overall prediction strength of the model. The strength of its influence is determined by its threshold (Schwarz et al., 2017). Specifically, *R*^2^ less than 0.3 means small impact size, 0.3–0.6 means medium impact size, and greater than 0.6 means large impact size. As seen in Table 4, the *R*^2^ of non-activist behaviors is 0.622, indicating a large impact size, and the *R*^2^ of environmental activism is 0.449, which shows a medium effect size (Schwarz et al., 2017). Next, the relative predictive relevance of the structural model was assessed by the Stone–Geisser criterion (*Q*^2^), derived through the blindfolding technique in PLS-SEM with an omission distance of 7 (Geisser, 1974; Stone, 1974). To be precise, *Q*^2^ between 0.02 and 0.15 means a small impact size, 0.15–0.35 is a medium impact size, and greater than 0.35 is a large impact size (Rigdon, 2014; Sarstedt et al., 2014). The *Q*^2^ of non-activist behaviors was 0.384, showing a large effect size. In addition, the *Q*^2^ of environmental activism was 0.256, showing a medium effect size.

**TABLE 4:** Fit indices for the four models in the study.

Endogenous latent constructs	R^2^	Q^2^
NA	0.622	0.384
EA	0.449	0.256

*NA, non-activist behaviors; EA, environmental activism.*

#### Path Relationship Evaluations

Table 5 shows the results of the hypothesis test. As expected, attitude (= 0.201, < 0.001), social norms (= 0.098, < 0.01), social trust (= 0.180, < 0.001), institutional trust (= 0.127, < 0.001), EI (= 0.175, < 0.001), and politicized identity (= 0.103, < 0.05) have positive effects on non-activist behaviors. Moreover, the impact of contextual constraints (= –0.161, < 0.001) on non-activist behaviors is significantly negative, thereby supporting H1a, H2a, H3a, H4a, H5a, H6a, and H7a.

**TABLE 5:** Results of algorithm and bootstrapping tests.

Hypothesis	β	T-value	*P-value*	Support
H1a: AT - > NA	0.201***	4.780	0.000	Yes
H1b: AT - > EA	0.127**	2.729	0.006	Yes
H2a: CC - > NA	–0.161***	4.757	0.000	Yes
H2b: CC - > EA	–0.130***	3.345	0.001	Yes
H3a: SN - > NA	0.098**	2.684	0.007	Yes
H3b: SN - > EA	0.204***	5.016	0.000	Yes
H4a: ST - > NA	0.180***	3.921	0.000	Yes
H4b: ST - > EA	0.172***	3.658	0.000	Yes
H5a: IT - > NA	0.127***	3.683	0.000	Yes
H5b: IT - > EA	–0.136***	3.601	0.000	No
H6a: EI - > NA	0.175***	4.064	0.000	Yes
H6b: EI- > EA	0.115*	2.434	0.015	Yes
H7a: PI - > NA	0.103*	2.410	0.016	Yes
H7b: PI - > EA	0.206***	4.364	0.000	Yes

*p < 0.05, **p < 0.01, ***p < 0.001.

*AT, attitude; SN, social norms; CC, contextual constraints; PI, politicized identity; EI, environmentalist self-identity; ST, social trust; IT, institutional trust; NA, non-activist behaviors; EA, environmental activism.*

Regarding to environmental activism, attitude (= 0.127, < 0.01), social norms (= 0.204, < 0.001), social trust (= 0.172, < 0.001), EI (β = 0.115, < 0.05), and politicized identity (= 0.206, < 0.001) are all positive predictors. As expected, contextual constraints (= –0.130, < 0.001) significantly affect environmental activism negatively. Thus, H1b, H2b, H3b, H4b, H6b, and H7b were confirmed, whereas institutional trust (= –0.136, < 0.001) has a negative impact on environmental activism, so H5b was not confirmed.

#### The Moderating Effect of Trust and Identity

Interaction indicators were added to the model, following Chin et al. (2003). The moderating effects of social trust and EI on the relationship between attitude and public-sphere PEB were significant, supporting H8a, H8b, H10a, and H10b. However, there was no significant evidence to prove the moderating effect of institutional trust on the relationship between attitude and public-sphere PEB. Thus, H9a and H9b were not supported. Meanwhile, the moderating role of politicized identity was supported partially. At the level of 5%, the moderating effect of politicized identity on attitude and environmental activism was significant (β = 0.092, p<0.01) but not significant on the relationship between attitude and non-activist behaviors, thereby supporting H11b but not H11a. Table 6 shows the moderating effects.

**TABLE 6:** Results of the moderating effect.

Moderator variable	Interacting	Dependent variable	β	*P-value*
ST	ST*AT	NA	0.098***	0.000
		EA	0.146***	0.000
IT	IT*AT	NA	0.040	0.380
		EA	0.052	0.500
EI	EI*AT	NA	0.143***	0.000
		EA	0.127**	0.002
PI	PI*AT	NA	0.050	0.304
		EA	0.092**	0.006

**p < 0.01, ***p < 0.001.

*AT, attitude; ST, social trust; IT, institutional trust; EI, environmentalist self-identity; PI, politicized identity; NA, non-activist behaviors; EA, environmental activism.*

## Discussion

With an extended Attitude-Behavior-Context theory, this study explored the impact of trust and identity on public-sphere PEB. At the same time, the moderating effects of trust and identity on the relationship between attitude and public-sphere PEB were examined. The main findings are as follows.

The results from our model show that trust is a significant determinant of public-sphere PEB. This finding is consistent with the previous literature (Arbuckle et al., 2013). Yet, social trust and institutional trust exhibited distinct effects on different types of public-sphere PEB. Specifically, social trust has almost the same effect on different public-sphere PEB. Social trust can make people believe that others will equally contribute to the environmental issues and reduce the free-rider problem in collective engagement (Atshan et al., 2020). Interestingly, the effects of institutional trust on the two types of public-sphere PEB are significantly different. In particular, institutional trust positively impacts non-activist behaviors, as expected. While contrary to the literature and our hypothesis, the impact of institutional trust on environmental activism is negative (Arbuckle et al., 2013). This finding may respond to Harring et al. (2019), who suggested that institutional trust has a certain “trust threshold” that could negatively affect cooperation. A possible explanation might be that institutional trust beyond a certain level makes people regard environmental activism as unnecessary, because they believed that the government could manage the related issue appropriately. This result also verified the finding of Dalton (2015), which implied that if the government established multiple ways for people to express their environmental activities, their environmental activism would decline.

Second, EI and politicized identity are significantly related to public-sphere PEB. In detail, compared with politicized identity, EI is a stronger predictor of non-activist behaviors but a weaker predictor of environmental activism. Specifically, politicized identity is more specific to collective action obligations (Alberici and Milesi, 2015), while EI is more prominent in taking environmental behaviors due to moral (van der Werff et al., 2013a) but less political reasons (Schmitt et al., 2019). Besides, environmental activism is a kind of environmental action based on solid collectivism, especially in the political field (Stern, 2000; Dalton, 2015) with a radical image label. Therefore, participating in this activity is driven more by people’s politicized identity than their environmentalist identity. This finding is consistent with the research of Schmitt et al. (2019), which proved that politicized identification is more predictive of activism. On the contrary, non-activist behaviors express a person’s environmental requirements more gently, emphasizing the perception of environmental responsibilities and obligations rather than collectivist action. Consequently, environmentalist self-identity has a more significant influence on non-activist behaviors than politicized identity.

Third, trust and identity are moderators of the relationship between attitude and public-sphere PEB. Particularly, the effect of attitude on public-sphere PEB is greater for residents with a high level of social trust. Social trust will encourage residents to translate their attitudes into practical actions. In the meantime, due to the mixed effect of institutional trust, its moderating effects are not significant in linking attitude and public-sphere PEB. Residents with high institutional trust believe that public-sphere PEB could efficiently help governments manage the environment, which induces the translation of their attitude. On the contrary, the higher institutional trust could also weaken people’s attitudes translation, because they feel no necessity to take action since the government can solve the problems properly (Harring et al., 2019). Moreover, EI significantly strengthens the effect of attitude on public-sphere PEB. That is, individuals would participate in either environmental activism or non-activist behaviors to affirm their identities or eliminate identity-related discomfort (Lacasse, 2016). Regarding politicized identity, the moderating effect on attitude and environmental activism is significantly positive but not significant on attitude and non-activist behaviors. A possible explanation is that environmental activism is a typical collective action with more visibility, strengthening people’s politicized identity and boosting their feelings of pride. Consequently, people with high politicized identity are more likely to pursue politicized satisfaction by participating in environmental activism. However, non-activist behaviors are not with political attributes or high visibility. Therefore, there was no difference in attitude transformation into non-activist behaviors between people with different degrees of politicized identity.

Finally, attitudes and social norms are positive factors of public-sphere PEB, while contextual constraints are negative predictors. These results are consistent with the existing research (Duarte et al., 2017). In addition, we further clarify that, compared to non-activist behaviors, attitudes had a weaker effect on environmental activism, while social norms had a stronger impact on environmental activism. A reasonable explanation might be that environmental activism is more characteristic of collective action, which requires support from others to participate in, compared to non-activist behaviors. Therefore, people need to consider social standards and the influence of others when they decide whether engage in environmental activism, except for their own attitudes. Furthermore, as a common belief held by the public (Ostrom, 2000), social norms might align self-interest with collective interests by imposing sanctions on individuals. The higher the degree of social norms, the more consistent people’s values and behavior standards, which will considerably boost environmental activism. However, non-activist behaviors’ participant decision is mainly based on individuals’ attitudes.

## Resource Identification Initiative

### Conclusion

This study explores the effects of trust and identity on Chinese residents’ public-sphere PEB based on an extended ABC theory. Our main findings indicate that social trust and institutional trust are significant predictors of residents’ public-sphere PEB through different mechanisms. Institutional trust positively impacts non-activist behaviors but negatively relates to environmental activism. Social trust has almost the same positive effect on the two types of public-sphere PEB. Also, social trust can enhance the impact of attitude on public-sphere PEB. Moreover, EI and politicized identity significantly relate to public-sphere PEB. Specifically, EI has a more substantial effect on non-activist behaviors than environmental activism, while the impact of politicized identity is just the opposite. Furthermore, EI strengthens the effect of attitude on public-sphere PEB. In addition, the moderating effect of politicized identity is significantly positive on attitude and environmental activism but not significant on attitude and non-activist behaviors.

### Theoretical Implications

This study makes contributions to the literature on PEB in three ways.

First, different from prior research, which has considered private-sphere or general PEB (Ertz et al., 2016; Wynes and Nicholas, 2017), this research focuses on public-sphere PEB and treats it as two constructs of non-activist behaviors and environmental activism, which is helpful to clarify the specific behavioral logic of public-sphere PEB.

Second, we extend the ABC theory by integrating trust and identity to provide a specific framework of public-sphere PEB, from the collective action perspective (Clayton and Opotow, 2003). As distinct from earlier findings, this study revealed that institutional trust negatively relates to environmental activism. It appears that, for residents with high trust in institutions, their environmental activism would decline.

Third, this study also offers a further explanation of how trust and identity influence public-sphere PEB adoption. Specifically, trust and identity impact public-sphere PEB participation directly and moderate the relationship between attitude and public-sphere PEB. In particular, we discuss trust from dimensions of social trust and institutional trust and consider EI and politicized identity as representatives of identity. This treatment helps to detangle the specific mechanisms of trust and identity on behaviors.

### Practical Implications

This study has several practical implications for governments and communities.

First, some strategies for building residents’ trust are necessary to promote their public-sphere PEB. To strengthen residents’ social trust, communities can organize community activities and invest in local social networks. For example, communities could create a website or social media platform (e.g., Weibo or WeChat) to connect to residents. Regarding institutional trust, the government could maintain a high degree of institutional trust of residents and motivate them to engage in more non-activist behaviors but less environmental activism, such as providing more information and public access to environmental matter, building a multi-directional dialog mechanism to increase the transparency of administrations, and guiding the public to participate in PEB appropriately. In this regard, a high degree of institutional trust may help residents to engage in non-activist behaviors. More importantly, the radical activism of public-sphere PEB could be transformed into a more institutionalized pattern of actions by the high institutional trust, at the same time.

Second, improving the identity of residents is a crucial way to motivate their participation in public-sphere PEB. Communities should motivate residents to join environmental groups and reward active participants of public-sphere PEB, such as writing policy proposals. These interventions will reinforce residents’ EI, and their motivation to engage in public-sphere PEB would increase, especially for non-activist behaviors. Furthermore, policymakers should provide more opportunities for residents to participate in public affairs to highlight their politicized identity, leading to more public-sphere PEB participants in return. Besides, communities could encourage environmental NGOs’ development and involvement in the policy process of public-sphere PEB, which is an important way to strengthen NGOs members’ identity. In addition, considering the role of identity, environmental policies must be shaped *via* group thinking rather than at an individual level.

Third, internal and contextual factors should not be neglected for the significant impacts of attitude, social norms, and contextual constraints. Some environmental campaigns and programs are necessary to enhance citizens’ attitudes and personal norms toward PEB, for example, explaining the environmental situation and the responsibility of citizens and highlighting the public-sphere PEB of their peers. As for contextual constraints, policymakers ought to undertake more structural and institutional reforms, letting residents have more voice and participation in public-sphere PEB.

### Limitations and Further Research

Some limitations should be considered. First, behaviors were assessed by an internet survey with self-reported measures. These methods may induce sample choosing bias and measurement errors. Mixed or experimental methods and actual behaviors data may be applied in the future. Second, cross-sectional data were used in this research, which could not capture the causal relationship between variables. Further studies could use longitudinal data. Finally, future research should explore the joint effects of contextual factors and attitudes on public-sphere PEB.

Future research may be developed from the following aspects. First, future exploration can consider whether the factors involved in this study have universal adaptability to public-sphere PEB in different cultural backgrounds. Trust and identity may have different effects on public-sphere PEB in different cultural contexts Therefore, their applicability to other cultural contexts requires further study. Second, future research could explore the mechanisms by which the factors involved in this study affect public-sphere PEB. It is helpful to further understand public-sphere PEB by clarifying some routines or mediators between trust, identity, and public-sphere PEB. Third, further research can use some combination of different theories to provide a more comprehensive framework for exploring multiple determinants of public-sphere PEB. Finally, the heterogeneity of individuals in their PEB participation is worthy of further discussion. For example, are different effects of trust and identity on public-sphere PEB between different gender and age individuals? or for different sociographical individuals, are there different determinants for private-sphere and public-sphere PEB?

## Data Availability Statement

The raw data supporting the conclusions of this article will be made available by the authors, without undue reservation.

## Ethics Statement

The studies involving human participants were reviewed and approved by the China Agricultural University. The patients/participants provided their written informed consent to participate in this study.

## Author Contributions

YX and ML conceptualized the theme, collected the data, analyzed the data, and wrote the first manuscript draft. YL reviewed and commented on the initial draft. All authors contributed to the article and approved the submitted version.

## Conflict of Interest

The authors declare that the research was conducted in the absence of any commercial or financial relationships that could be construed as a potential conflict of interest.

## Publisher’s Note

All claims expressed in this article are solely those of the authors and do not necessarily represent those of their affiliated organizations, or those of the publisher, the editors and the reviewers. Any product that may be evaluated in this article, or claim that may be made by its manufacturer, is not guaranteed or endorsed by the publisher.

## Footnotes

1
https://www.wjx.cn/
