# Supplementary material for: Trust, Identity, and Public-Sphere Pro-environmental Behavior in China: An Extended Attitude-Behavior-Context Theory
Source: Front Psychol. 2022 Jun 24;13:919578. doi: 10.3389/fpsyg.2022.919578 (PMC9266346; doi:10.3389/fpsyg.2022.919578)
Supplement: Supplementary file 1 [file Table_1.pdf]

Supplementary Material

1 Supplementary Figures and Tables

1.1 Supplementary Tables

Appendix A Demographic profile of respondents.

Characteristic	Demographic	Frequency	Percentage(%)
Gender	Female	456	56.8
	Male	347	43.2
Marital status	Single	287	35.7
	Married	516	64.3
Age(years)	18-25	175	21.8
	26-35	425	52.9
	36-45	155	19.3
	46 and above	48	6.0
Educational	Senior high or below	56	7.0
	Associate degree	136	16.9
	Bachelor degree	532	66.3
	Master's degree above	79	9.8
Income(monthly)	Less than ￥5000($705)	156	19.4
	￥5000($705)-￥10000($1410)	330	41.1
	￥10000($1410)-￥15000($2115)	204	25.4
	￥15000($2115)-￥20000($2820)	85	10.6
	More than ￥20000($2820)	28	3.5
province	Beijing	123	15.3
	Shanghai	149	18.6
	Guangdong	255	31.8
	Jiangsu	138	17.2
	Zhejiang	138	17.2
area	Inland areas	475	59.2
	Coastal areas	328	40.8
	Total	803	100

Appendix B Questionnaire observed variables.

Latent variables	Items no.	Questions	Literature sources
Environmental activism	EA1	How often do you participate in protests against current environmental conditions?	Stern (2000)；Postmes (2002)；Jiménez-Castillo and Ortega-Egea, (2015)
	EA2	How often do you participate in petitions demanding an improvement of government policies regarding the environment?	
	EA3	How often do you participate in a group action, such as a march or rally, in support of efforts to stop environmental pollution.	
	EA4	How often do you give financial support to an environmental group？	
Non-activist behaviours	NA1	How often do you get involved with any groups whose main aim is to preserve or protect the environment?”	Dono et al. (2010) ；Ertz et al. (2016)
	NA2	How often do you vote for a government proposing environmentally conscious policies? (DELETE)	
	NA3	How often do you share posts about the environment on social media?	
	NA4	How often do you write a letter or called your government official to support strong environmental protection?	
Environmentalist self-identity	EI1	Acting environmentally friendly is an important part of who I am. (DELETE)	Whitmarsh and O’Neill (2010); Fielding et al. (2008)
	EI2	I think of myself as someone who is very concerned with environmental issues.	
	EI3	I would describe myself as environmentally responsible.	
	EI4	I see myself as an environmentally-friendly person.	
Politicised identity	PI1	I am an activist in community affairs.	Van Zomeren et al. (2008)
	PI2	I feel strong ties with the community I live. (DELETE)	
	PI3	I see myself as a member of the community I live.	
Attitude	AT1	I think it is useful to behave pro-environmentally.	Gao et al. (2017); Gkargkavouzi, et al. (2019).
	AT2	I think behaving in an environmentally responsible way to save energy is a wise action.	
	AT3	Adoption of eco-friendly behavior is valuable to alleviate nature resources shortage	
Social Trust	ST1	Residents in this area are trustworthy and reliable.	Kuo et al., (2021); Liu et
	ST2	When I have difficulties, my neighbors are always willing to help me.	

	ST3	I think the community representatives are trustworthy.	al.(2014)
	ST4	Most people in society are honest and reliable. (DELETE)	
Institutional trust	IT1	I can rely on the government to deal with the environmental matters fairly.	Kitt et al., (2021); Caferra et al., (2021)
	IT2	I feel confident that political system allows people to have influence on environmental protection matters.	
	IT3	I believe that the government intends to act the best interests of the public, when designing environment-related policies.	
Social norms	SN1	Family members whose opinion I value would approve of my engagement in public sphere PEB.	Ling and Xu (2020)
	SN2	Close friends who are important to me would support my engagement in public sphere PEB.	
	SN3	The residents in my community would support my engagement in public sphere PEB.	
	SN4	The general public would endorse my engagement in public sphere PEB.(DELETE)	
Context constraints	CC1	It is expensive to engage in public sphere PEB. (DELETE)	Gkargkavouzi et al. (2019); Ertz et al. (2016).
	CC2	One needs time to engage in public sphere PEB.	
	CC3	Needed effort makes the engagement in engage in public sphere PEB.	
	CC4	Limited opportunity of engaging in public sphere PEB.	

1.2 Supplementary Figures

Appendix C Slope analyses of moderating effect

Moderating effect of ST on AT and NA

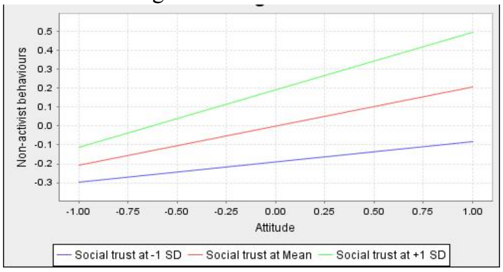

Moderating effect of ST on AT and EA

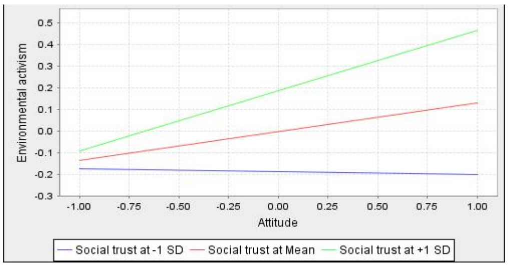

Moderating effect of EI on AT and NA

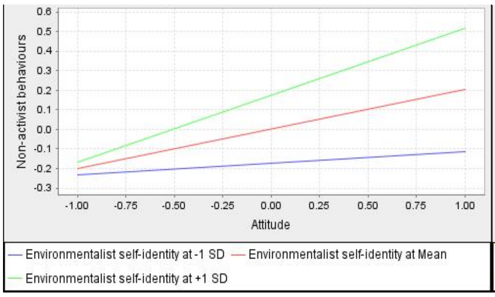

Moderating effect of EI on AT and EA

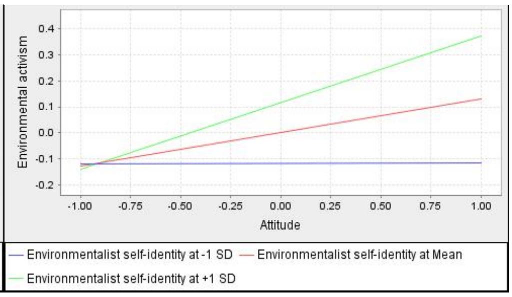

Moderating effect of PI on AT and EA

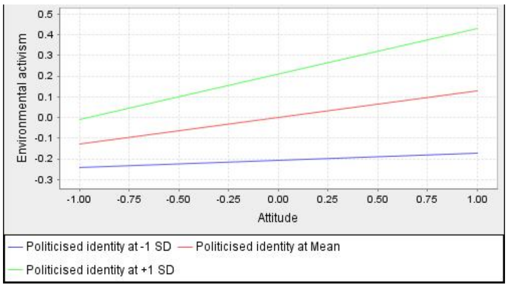

AT, attitude; ST, Social trust; EI, Environmentalist self-identity; PI, Politicized identity; NA, Non-activist behaviors; EA, Environmental activism.
